# Supplementary material for: Morquio A Syndrome: Identification of Differential Patterns of Molecular Pathway Interactions in Bone Lesions
Source: Int J Mol Sci. 2024 Mar 12;25(6):3232. doi: 10.3390/ijms25063232 (PMC10970612; doi:10.3390/ijms25063232)
Supplement: Supplementary file 1 [file ijms-25-03232-s001.zip › Supplemental data legends.pdf]

Supplementary Table S1: This table lists all proteins identified by DDA (Sheet1), along with the corresponding Uniprot code and gene and protein names. Sheets 2 and 3 list proteins detected exclusively in WT or UNT mice. The remaining sheets show the detected proteins categorized according to function, together with the corresponding Uniprot code, gene and protein name, and the results of the DDA analysis (indicating whether proteins were detected in WT and/or UNT mice).

Supplementary Table S2: The mean MS peak area for each protein was determined for each sample, and the averaged total area of all transitions for each protein compared (Student's t-test; MarkerView software, Sciex, Redwood City, CA, USA). The resulting p-value indicates the capacity of each variable to distinguish between the two groups. Table shows protein name, m/z, mouse group (UNT or WT), use, t-value, p-value, log(p), mean (UNT and WT,) mean value in UNT mice and in WT mice, sigma UNT, sigma WT, Delta, fold change, and Log (fold change).

Supplementary Table S3: Table shows all dysregulated proteins, based on the results of the SWATH-MS analysis (P>0.05 and a FC>1 [upregulated in UNT mice] or FC<1 [upregulated in WT mice]). Proteins are grouped according to function.

Supplementary Table S4: Results of quantitative protein analysis. Table shows DEPs as detected by SWATH analysis (Sheet 1), together with the corresponding Uniprot code, gene and protein name, p-value (Student t-test) and fold change (FC); and by spectral count analysis (Sheet 2), together with the corresponding protein name, p-value (Fisher's exact test) and fold change (FC).

Supplementary Table S5: Table shows all proteins identified by DDA analysis in WT (Sheet 1) and UNT (Sheet 2) mice, together with the corresponding Uniprot code, peptides (95%) and percentage sequence coverage (% Cov).

Supplementary Table S6: Table lists all proteins identified in the SWATH-MS analysis library, together with corresponding Uniprot code, peptides (95%) and percentage sequence coverage (% Cov).

Supplementary Table S7: List of proteins identified in the in silico validation. A) Comparison between mouse DEPs and previous findings using human samples. B) Comparison between mouse DEPs and other data sets corresponding to human bone and bone marrow samples.

Supplementary Table S8: List of proteins identified in the SWATH-MS analysis that interact with Ldha or Ldhb. The table shows the following information: protein code, gene name, names of proteins interacting with Ldha or Ldhb, and SWATH fold changes (FC).

Supplementary Figure S1: A) Venn diagram showing the number of proteins detected by DDA in the two studied groups (all proteins have a < *1% FDR)*. B) Volcano plot depicting the results of the SWATH-MS quantitative proteomics analysis. Blue dots represent proteins for which significant differences in expression were observed between groups (p <0.05 and FC >1.4).
