# Supplementary material for: Morquio A Syndrome: Identification of Differential Patterns of Molecular Pathway Interactions in Bone Lesions
Source: Int J Mol Sci. 2024 Mar 12;25(6):3232. doi: 10.3390/ijms25063232 (PMC10970612; doi:10.3390/ijms25063232)
Supplement: Supplementary file 1 [file ijms-25-03232-s001.zip › supplementary Material and methods_rev.pdf]

Morquio A Syndrome: Identification of Differential Patterns of Molecular Pathway Interactions in Bone Lesions

J. Victor. Álvarez [1,2], Susana B. Bravo [3], María Pilar Chantada-Vázquez [3], Carmen Pena [3], Cristóbal Colón [1,2], Shunji Tomatsu [4], Francisco J. Otero-Espinar [5,6] and María L. Couce [1,2,*]

Quantitative Proteomic analysis by TripleTOF 6600 LC-MSMS System

Protein digestion

In order to perform global protein identification and quantification, an equal amount of protein (over 100 ug), from mouse bone samples (from 4 UNT and 4 WT mice) was loaded on a 10% SDS-PAGE gel. The run was stopped as soon as the front had penetrated 3 mm into the resolving gel (Bonzon-Kulichenko et al., 2011; Perez-Hernandez et al., 2013). The protein bands were detected by Sypro Ruby fluorescent staining (Lonza, Switzerland), excised, and processed for in-gel, manual tryptic digestion as described elsewhere (Shevchenko et al., 1996). Gel pieces were reduced with 10 mM dithiothreitol (Sigma-Aldrich, St. Louis, MO) in 50 mM ammonium bicarbonate (Sigma-Aldrich, St. Louis, MO) and alkylated with 55 mM iodoacetamide (Sigma- Aldrich, St. Louis, MO) in 50 mM ammonium bicarbonate. Then, gel pieces were rinsed with 50 mM ammonium bicarbonate in 50% methanol (HPLC grade, Scharlau, Barcelona, Spain), dehydrated by addition of acetonitrile (HPLC grade, Scharlau, Barcelona, Spain), and dried in a SpeedVac. Modified porcine trypsin (Promega, Madison, WI, USA) was added to the dry gel pieces at a final concentration of 20 ng/µl in 20mM ammonium bicarbonate, incubating them at 37 °C for 16 h. Peptides were extracted thrice by 20 min incubation in 40 µL of 60% acetonitrile in 0.5% HCOOH. The resulting peptide extracts were pooled, concentrated in a SpeedVac, and stored at −20 °C.

Mass spectrometric analysis (DDA acquisition)

Digested peptides (over 4μg of each sample) were separated using Reverse Phase Chromatography. A gradient was created using a micro liquid chromatography system (Eksigent Technologies nanoLC 400, Sciex) coupled to a high-speed Triple TOF 6600 mass spectrometer (Sciex) with a micro flow source. The analytical column was a silica-based reversed phase column Eksigent C18 150 × 0.30 mm, 3 mm particle size and 120 Å pore size (Eksigent, Sciex). The trap column was a YMC-TRIART C18 (YMC Technologies, Teknokroma) with a 3 mm particle size and 120 Å pore size, switched on-line with the analytical column. The loading pump delivered a solution of 0.1% formic acid in water at 10 µl/min. The micro-pump generated a flow-rate of 5 µl/min and was operated under gradient elution conditions, using 0.1% formic acid in water as mobile phase A, and 0.1% formic acid in acetonitrile as mobile phase B. Peptides were separated using a 90 minutes gradient ranging from 2% to 90% mobile phase B.

Data acquisition was performed by a TripleTOF 6600 System (Sciex, Foster City, CA) using a data-dependent analysis (DDA) workflow. Source and interface conditions were the following: ionspray voltage floating (ISVF) 5500 V, curtain gas (CUR) 25, collision energy (CE), 10 and ion source gas 1 (GS1) 25. Instrument was operated with Analyst TF 1.7.1 software (Sciex, USA). Switching criteria was set to ions greater than mass to charge ratio (m/z) 350 and smaller than m/z 1400 with charge state of 2–5, mass tolerance of 250 ppm and an abundance threshold of more than 200 counts per second (cps). Previous target precursor ions were excluded for 15 s. The instrument was automatically calibrated every 4 hours using tryptic peptides from PepCalMix as external calibrant.

Data analysis

After MSMS analysis, data files were processed using ProteinPilot™ 5.0.1 software from Sciex, which uses the algorithm Paragon™ for database search and Progroup™ for data grouping. Data was searched using a Mouse specific Uniprot database (https://www.uniprot.org/uniprotkb?query=mus%20musculus

(release 2023_03, 21957 mouse proteins), specifying iodoacetamide at cysteine alkylation as variable modification and methionine oxidation as fixed modification. False discovery rate was performed using a non-lineal fitting method, displaying only those results that reported a 1% Global false discovery rate or better. (Shilov et al., 2007)

Relative quantification: Spectral count

Scaffold (version Scaffold-5.2.2 Proteome Software Inc., Portland, OR) was used to validate MSMS based peptide and protein identifications and to perform a semiquantitative analysis by spectral counting (Lee et al., 2019; López-López et al., 2022). Peptide identifications were accepted if they could be established at greater than 97.0% probability to achieve a false discovery rate (FDR) <1.0% by the Scaffold Local FDR algorithm. Protein identifications were accepted if they could be established at greater than 99.0% probability to achieve a FDR <1.0% and contained at least 2 identified peptides. Protein probabilities were assigned by the Protein Prophet algorithm. The Fisher's exact test was used for quantification and fold change determination. Proteins were considered differentially expressed when $P<0.05$. It should be noted that to quantify changes in spectral counts, we estimated -fold changes as proposed by Beissbarth et al. (Beißbarth et al., 2004) for serial analysis of gene expression (SAGE) data, which avoids the discontinuity seen in simple count ratios when a protein shows spectral count 0 in one of the samples. Proteins with a FC=0 were considered present only in UNT mice, while those with a FC=Infinity were considered present exclusively in WT mice.

Regarding validation and comparison, both quantitative techniques (spectral count and SWATH-MS) are able to give a protein change between groups. Indicated that in the spectral count quantification several fold change of 1 was observed join at a p-value increase This trend is expected and supports the validity of the results obtained. Spectral count values were fully convergent with those found by SWATH-

MS, but with lower p values and higher FC values. These differences between the two approaches are due to the much larger fold change resulting from the MSMS analysis (Freund & Prenni, 2013).

Protein quantification by SWATH-MS (Sequential Window Acquisition of all Theoretical Mass Spectra)

Since its early development, SWATH-MS has proved a valuable translational tool in multiple fields, and has been successfully applied by our group in a wide range of studies (Álvarez et al., 2020; Chantada-Vázquez et al., 2021; JV et al., 2021; M et al., 2021; Novelle et al., 2021; Tamara et al., 2020, 2021)

Generation of the spectral library

To build the MSMS spectral libraries, peptide solutions were analyzed by a shotgun data-dependent acquisition (DDA) approach using micro-LC-MSMS. To ensure adequate representation of the peptides and proteins present in all samples, pooled vials of samples from each group were prepared using equal volumes from the original samples. For each pool (UNT and WT), 4 µL was separated into a micro-LC system Ekspert nLC425 (Eksigen, Dublin, CA, USA) using an Eksigent C18 150 × 0.30 mm, 3 mm particle size and 120 Å pore size (Eksigent, Sciex), at a flow rate of 5 µL/min. Water and ACN, both containing 0.1% formic acid, were used as solvents A and B, respectively. The gradient run consisted of 5–95% of B for 30 min, 90% of B for 5 min, and finally 5% of B for 5 min for column equilibration (total run time, 40 min). As the peptides eluted, they were directly injected into a hybrid quadrupole-TOF mass spectrometer Triple TOF 6600 (Sciex, Redwood City, CA, USA) operated with a data-dependent acquisition system in positive ion mode. A Micro source (Sciex) was used for the interface between microLC and MS, with application of 2600 V. The acquisition mode consisted of a 250 ms survey MS scan from 400–1250 m/z, followed by a MSMS scan from 100–1500 m/z (25 ms acquisition time) of the top 65 precursor ions from the survey scan, with a total cycle time of 2.8 s. The fragmented precursors were then added to a dynamic exclusion list for 15 s. Any singly charged ions were excluded from the MSMS analysis.

Peptide and protein identifications were performed using Protein Pilot software (version 5.0.1, Sciex). Data were analyzed using a Mouse specific Uniprot database (https://www.uniprot.org/uniprotkb?query=mouse) (release 2023_03, 21957 mouse proteins), specifying iodoacetamide as Cys alkylation as variable modification and methionine oxidation as fixed modification. The false discovery rate (FDR) was set to 1% for both peptides and proteins. The MSMS spectra of the identified peptides were then used to generate the spectral library for SWATH-MS peak extraction using an add-in for PeakView Software (version 2.2, Sciex): MSMS^{ALL} with SWATH-MS Acquisition MicroApp (version 2.0, Sciex). Peptides with a confidence score >99% (obtained from the Protein Pilot database search) were included in the spectral library.

Relative quantification by SWATH-MS acquisition

SWATH–MS (Sequential Window Acquisition of all Theoretical Mass Spectra) acquisition was performed on a TripleTOF® 6600 LC-MSMS system (Sciex). Technical triplicates of peptides mixtures (4 µL) from each individual bone sample (4 Bone WT and 4 Bone UNT) were analyzed using a data-independent acquisition (IDA) method (12 samples in total). Each 4-µL sample was analyzed with the LC-MSMS equipment, applying the aforementioned LC gradient used to build the spectral library, but using the SWATH-MS acquisition method instead. The method involved repeating a cycle that consisted of the acquisition of 100 TOF MSMS scans (400–1500 m/z, high sensitivity mode, 50 ms acquisition time) of overlapping sequential precursor isolation windows of variable width (1 m/z overlap), covering the 400–1250 m/z mass range with a previous TOF MS scan (400–1500 m/z, 50 ms acquisition time) for each cycle. Total cycle time was 6.3 s The 100 variable windows width was optimized according to the ion density found in the DDA runs using a SWATH-MS variable window calculator worksheet from Sciex.

Data analysis

The targeted data extraction of the fragment ion chromatogram traces from the SWATH-MS runs was performed by PeakView (version 2.2, Sciex) using the SWATH-MS Acquisition MicroApp (version 2.0). This application processed the data using the spectral library generated from the shotgun data. Up to 10 peptides per protein and 7 fragments per peptide were selected, based on signal intensity; any shared and modified peptides were excluded from the processing. Five-minute windows and 30 ppm widths were used to extract the ion chromatograms; SWATH-MS quantization was attempted for all proteins in the ion library that were identified by ProteinPilot™ 5.0.1 with a FDR <1%. The retention times of the peptides that were selected for each protein were realigned in each run according to the iRT (internal retention time) peptides corresponding to the different proteins identified in each sample and eluted along the whole-time axis. The chromatograms of the extracted ions were then generated for each selected fragment ions. Peak areas for the protein were obtained by summing the peak areas from 10 peptides (MS scan) and 7 corresponding fragment ions (MSMS scan) from each peptide. PeakView 2.2 computed a FDR and a score for each assigned peptide according to the chromatographic and spectra components. Only peptides with a FDR <5% were used for protein quantization. Protein quantization was performed by adding the peak areas of the corresponding peptides.

The integrated peak areas (processed. mrkvw files from PeakView 2.2) were directly exported to the MarkerView software (version 1.3.1, Sciex) for relative quantitative analysis. The export generated 3 files containing quantitative information about individual ions, the summed intensity of different ions for a particular peptide, and the summed intensity of different peptides for a particular protein. MarkerView 1.3.1 uses processing algorithms that accurately find chromatographic and spectral peaks directly from the raw SWATH-MS data. Data alignment by MarkerView 1.3.1 compensates for minor variations in both mass and retention time values, ensuring that identical compounds in different samples are accurately compared to one another. Unsupervised multivariate statistical analysis using principal component analysis was performed to compare the data from the different samples. A most-like ratio normalization

was performed after statistical analysis to control for possible uneven sample loss across the different samples during the sample preparation process (Lambert et al., 2013; Redestig et al., 2009). The average MS peak area for each protein was derived from each sample, followed by analysis using a Student's t-test (MarkerView 1.3.1 software, Sciex, Redwood City, CA, USA) to compare between samples based on the averaged total area of all transitions for each protein. The t-test result (p-value) indicates how well each variable distinguishes between the two groups. Candidate proteins were selected for each library based on t-test results ($p < 0.05$ and FC (increase or decrease) >1.5 or <0.6).

Proteomic analysis quality control

The proteomic analyzes are always carried out with rigorous quality controls. Thus, every 4 hours the triple TOF 6600 is calibrated with a mixture of commercial external peptides (Pepcalmix sciex) In addition, our quality requirements mean that every 1-2 weeks the equipment is tuned using Sciex's Tunning solution, which consists of a single peptide with which the equipment adjusts numerous parameters, including the lenses that focus the peptides towards the detector and the detector. Another quality control is performed by analyzing before each very large group of samples a known sample, in our case an extract of human cells (K562) digested with trypsin, also commercial. Carrying out this analysis allows us to know the condition in which the equipment is found, since in a DDA analysis the sensitivity of the equipment must allow the identification of more than 1900 proteins in this extract.

Functional and pathway analysis

We conducted pathway analysis using Reactome (https://reactome.org/), which uses a statistical (hypergeometric distribution) test that determines whether certain pathways are over-represented (enriched) and produces a probability score, which is corrected for FDR using the Benjamini–Hochberg

method. Most significant pathways were represented using Reactome pathway diagrams. Protein interactions were evaluated using String (https://string-db.org/), considering a minimum required interaction score of PPI=0.9 (protein protein interaction) and a FDR <0.05. Venn diagrams were generated using http://www.interactivenn.net/ and box plots using GraphPad Prism 9. Statistical analyses were performed using MarkerView 1.3.1 or Scaffold software. Volcano plots and box plots were generated using GraphPad Prism 9 (GraphPad Software, San Diego, CA, USA).

Álvarez, V. J., Bravo, S. B., Chantada-Vazquez, M. P., Colón, C., De Castro, M. J., Morales, M., Vitoria, I., Tomatsu, S., Otero-Espinar, F. J., & Couce, M. L. (2020). Characterization of New Proteomic Biomarker Candidates in Mucopolysaccharidosis Type IVA. *International Journal of Molecular Sciences*, *22*(1), 226. https://doi.org/10.3390/ijms22010226

Beißbarth, T., Hyde, L., Smyth, G. K., Job, C., Boon, W. M., Tan, S. S., Scott, H. S., & Speed, T. P. (2004). Statistical modeling of sequencing errors in SAGE libraries. *Bioinformatics (Oxford, England)*, *20 Suppl 1*(SUPPL. 1). https://doi.org/10.1093/BIOINFORMATICS/BTH924

Bonzon-Kulichenko, E., Pérez-Hernández, D., Núñez, E., Martínez-Acedo, P., Navarro, P., Trevisan-Herraz, M., Ramos, M. D. C., Sierra, S., Martínez-Martínez, S., Ruiz-Meana, M., Miró-Casas, E., García-Dorado, D., Redondo, J. M., Burgos, J. S., & Vázquez, J. (2011). A robust method for quantitative high-throughput analysis of proteomes by 18O labeling. *Molecular & Cellular Proteomics : MCP*, *10*(1), M110.003335. https://doi.org/10.1074/mcp.M110.003335

Chantada-Vázquez, M. del P., García Vence, M., Serna, A., Núñez, C., & Bravo, S. B. (2021). SWATH-MS Protocols in Human Diseases. In *Methods in Molecular Biology* (Vol. 2259, pp. 105–141). Humana Press Inc. https://doi.org/10.1007/978-1-0716-1178-4_7

Freund, D. M., & Prenni, J. E. (2013). Improved detection of quantitative differences using a combination of spectral counting and MS/MS total ion current. *Journal of Proteome Research*, *12*(4), 1996–2004. https://doi.org/10.1021/PR400100K

JV, Á., SB, B., MP, C.-V., S, B.-G., C, C., O, L.-S., S, T., FJ, O.-E., & ML, C. (2021). Plasma Proteomic Analysis in Morquio A Disease. *International Journal of Molecular Sciences*, *22*(11). https://doi.org/10.3390/IJMS22116165

Lambert, J. P., Ivosev, G., Couzens, A. L., Larsen, B., Taipale, M., Lin, Z. Y., Zhong, Q., Lindquist, S., Vidal, M., Aebersold, R., Pawson, T., Bonner, R., Tate, S., & Gingras, A. C. (2013). Mapping differential interactomes by affinity purification coupled with data-independent mass spectrometry acquisition. *Nature Methods*, *10*(12), 1239–1245. https://doi.org/10.1038/NMETH.2702

Lee, H. Y., Kim, E. G., Jung, H. R., Jung, J. W., Kim, H. B., Cho, J. W., Kim, K. M., & Yi, E. C. (2019). Refinements of LC-MS/MS Spectral Counting Statistics Improve Quantification of Low Abundance Proteins. *Scientific Reports 2019 9:1*, *9*(1), 1–10. https://doi.org/10.1038/s41598-019-49665-1

López-López, M., Regueiro, U., Bravo, S. B., Chantada-Vázquez, M. del P., Pena, C., Díez-Feijoo, E., Hervella, P., & Lema, I. (2022). Shotgun Proteomics for the Identification and Profiling of the Tear Proteome of Keratoconus Patients. *Investigative Ophthalmology & Visual Science*, *63*(5). https://doi.org/10.1167/IOVS.63.5.12

M, G.-V., MDP, C.-V., A, S.-F., R, A., A, B. de la I., A, O.-G., M, G.-G., JM, C.-T., C, N., JJ, B., & SB, B. (2021). Protein Extraction From FFPE Kidney Tissue Samples: A Review of the Literature and Characterization of Techniques. *Frontiers in Medicine*, *8*. https://doi.org/10.3389/FMED.2021.657313

Novelle, M. G., Bravo, S. B., Deshons, M., Iglesias, C., García-Vence, M., Annells, R., da Silva Lima, N., Nogueiras, R., Fernández-Rojo, M. A., Diéguez, C., & Romero-Picó, A. (2021). Impact of liver-specific GLUT8 silencing on fructose-induced inflammation and omega-oxidation. *IScience*, 102071.

https://doi.org/10.1016/j.isci.2021.102071
Perez-Hernandez, D., Gutiérrez-Vázquez, C., Jorge, I., López-Martín, S., Ursa, A., Sánchez-Madrid, F., Vázquez, J., & Yáñez-Mó, M. (2013). The intracellular interactome of tetraspanin-enriched microdomains reveals their function as sorting machineries toward exosomes. *The Journal of Biological Chemistry*, *288*(17), 11649–11661. https://doi.org/10.1074/jbc.M112.445304

Redestig, H., Fukushima, A., Stenlund, H., Moritz, T., Arita, M., Saito, K., & Kusano, M. (2009). Compensation for systematic cross-contribution improves normalization of mass spectrometry based metabolomics data. *Analytical Chemistry*, *81*(19), 7974–7980. https://doi.org/10.1021/AC901143W/SUPPL_FILE/AC901143W_SI_001.PDF

Shevchenko, A., Wilm, M., Vorm, O., & Mann, M. (1996). Mass Spectrometric Sequencing of Proteins from Silver-Stained Polyacrylamide Gels. *Analytical Chemistry*, *68*(5), 850–858. https://doi.org/10.1021/ac950914h

Shilov, I. V., Seymour, S. L., Patel, A. A., Loboda, A., Tang, W. H., Keating, S. P., Hunter, C. L., Nuwaysir, L. M., & Schaeffer, D. A. (2007). The Paragon Algorithm, a Next Generation Search Engine That Uses Sequence Temperature Values and Feature Probabilities to Identify Peptides from Tandem Mass Spectra. *Molecular & Cellular Proteomics*, *6*(9), 1638–1655. https://doi.org/10.1074/mcp.T600050-MCP200

Tamara, C., Nerea, L.-B., Belén, B. S., Alberto, M.-V., Aurelio, S., Iván, C., Javier, B., Felipe, C. F., & María, P. (2021). HUMAN OBESE WHITE ADIPOSE TISSUE SHEDS DEPOT-SPECIFIC EXTRACELLULAR VESICLES AND REVEALS CANDIDATE BIOMARKERS FOR MONITORING OBESITY AND ITS COMORBIDITIES. *Translational Research*. https://doi.org/10.1016/j.trsl.2021.01.006

Tamara, C., Nerea, L.-B., Belén, B. S., Aurelio, S., Iván, C., Fernando, S., Javier, B., Felipe, C. F., & María, P. (2020). Vesicles Shed by Pathological Murine Adipocytes Spread Pathology: Characterization and Functional Role of Insulin Resistant/Hypertrophied Adiposomes. *International Journal of Molecular Sciences*, *21*(6). https://doi.org/10.3390/ijms21062252
