# Supplementary material for: Morquio A Syndrome: Identification of Differential Patterns of Molecular Pathway Interactions in Bone Lesions
Source: Int J Mol Sci. 2024 Mar 12;25(6):3232. doi: 10.3390/ijms25063232 (PMC10970612; doi:10.3390/ijms25063232)
Supplement: Supplementary file 1 [file ijms-25-03232-s001.zip › Supplementary Table S3.pdf]

Supplementary Table S3

(FC>1 elevated in UNT group, FC<1 elevated in WT group)

Section S1.1. Glycolysis, male UNT vs male WT mice

Protein	Code	Name	SWATH	
			P_value	FC
P06745	Gpi	Glucose-6-phosphate isomerase	0.0043	3.2287
P05064	Aldoa	Fructose-bisphosphate aldolase A	0.0375	18.7808
P13707	Gpd1	Glycerol-3-phosphate dehydrogenase [NAD(+)], cytoplasmic	0.0520	1.4896
Q9D0F9	Pgm1	Phosphoglucomutase-1	0.0205	1.9249
P17182	Eno1	Alpha-enolase	0.0121	0.2793
P21550	Eno3	Beta-enolase	0.0525	4.5256
P06151	Ldha	L-lactate dehydrogenase A chain	0.0434	9.0581
P16125	Ldhb	L-lactate dehydrogenase B chain	0.0089	2.5319

Section S1.2. TCA. male UNT vs male WT mice

Protein	Code	Name	SWATH	
			P_value	FC
Q9D051	Pdhb	Pyruvate dehydrogenase E1 component subunit beta, mitochondrial	0.0362	0.3913
Q9D6R2	Idh3a	Isocitrate dehydrogenase [NAD] subunit alpha, mitochondrial	0.0144	3.4270
Q8K2B3	Sdha	Succinate dehydrogenase [ubiquinone] flavoprotein subunit, mitochondrial	0.0327	2.6715
P08249	Mdh2	Malate dehydrogenase, mitochondrial	0.0426	19.0472

Section S1.3. OXPHOS, male UNT vs maleWT

Protein	Code	Name	SWATH	
			P_value	FC
Complex I				
Q9CQZ5	Ndufa6	NADH dehydrogenase [ubiquinone] 1 alpha	0.0246	86.5140

		subcomplex subunit 6		
Q9ERS2	Ndufa13	NADH dehydrogenase [ubiquinone] 1 alpha subcomplex subunit 13	0.0166	8.1749
Q9CQJ8	Ndufb9	NADH dehydrogenase [ubiquinone] 1 beta subcomplex subunit 9	0.0246	3.3900
Q9CQ54	Ndufc2	NADH dehydrogenase [ubiquinone] 1 subunit C2	0.0452	3.5213
Q91VD9	Ndufs1	NADH-ubiquinone oxidoreductase 75 kDa subunit, mitochondrial	0.0199	2.0976
Q91YT0	Ndufv1	NADH dehydrogenase [ubiquinone] flavoprotein 1, mitochondrial	0.0340	3.9303
Protein	Code	Name	SWATH	
			P_value	FC
Complex II				
Q8K2B3	Sdha	Succinate dehydrogenase [ubiquinone] flavoprotein subunit, mitochondrial	0.0327	2.6715
Q9CQA3	Sdhb	Succinate dehydrogenase [ubiquinone] iron-sulfur subunit, mitochondrial	0.0028	14.5768
Complex III				
Q9CR68	Uqcrfs1	Cytochrome b-c1 complex subunit Rieske, mitochondrial	0.0552	1.5274
		Complex IV		
P12787	Cox5a	Cytochrome c oxidase subunit 5A, mitochondrial	0.0106	5.6382
P43023	Cox6a2	Cytochrome c oxidase subunit 6A2, mitochondrial	0.0177	5.1320
P56391	Cox6b1	Cytochrome c oxidase subunit 6B1	0.0034	2.5879
P48771	Cox7a2	Cytochrome c oxidase subunit 7A2, mitochondrial	0.0359	5.1496
ATP synthase Complex V				
Q9D3D9	Atp5f1d	ATP synthase subunit delta, mitochondrial	0.0039	5.1416
P56135	Atp5mf	ATP synthase subunit f, mitochondrial	0.0369	12.1206
Q9CQQ7	Atp5pb	ATP synthase F(0) complex subunit B1, mitochondrial	0.0228	2.1821
Q9DCX2	Atp5pd	ATP synthase subunit d, mitochondrial	0.0017	2.1766
Q9DB20	Atp5po	ATP synthase subunit O, mitochondrial	0.0309	6.7524
Q78IK2	Atp5mk	ATP synthase membrane subunit K, mitochondrial	0.0457	0.2169

Section S1.4. Beta oxidation, male UNT vs male WT

Protein	Code	Name	SWATH	
			P_value	FC

P50544	Acadvl	Very long-chain specific acyl-CoA dehydrogenase, mitochondrial	0.0226	3.4695
P42125	Eci1	Enoyl-CoA delta isomerase 1, mitochondrial	0.0023	0.6253
P11404	Fabp3	Fatty acid-binding protein, heart	0.0490	33.7581

Section S2.1. ROS, male UNT vs male WT

Protein	Code	Name	SWATH	Protein
			P_value	FC
P08228	Sod1	Superoxide dismutase [Cu-Zn]	0.0160	9.8360
P35700	Prdx1	Peroxiredoxin-1	0.0069	3.4018
Q61171	Prdx2	Peroxiredoxin-2	0.0051	8.0147
P20108	Prdx3	Thioredoxin-dependent peroxide reductase, mitochondrial	0.0265	24.7061
P99029	Prdx5	Peroxiredoxin-5	0.0263	6.4456
P27005	S100a8	Protein S100-A8	0.0062	2.9169
P31725	S100a9	Protein S100-A9	0.0300	17.3367

Section S2.2. Iron transport, male UNT vs male WT

Protein	Code	Name	SWATH	
			P_value	FC
Q921I1	Tf	Serotransferrin	0.004	4.455
Q91WS0	Cisd1	CDGSH iron-sulfur domain-containing protein 1	0.023	7.278
P09528	Fth1	Ferritin heavy chain	0.029	17.2605
P04247	Mb	Myoglobin	0.0455	33.4430
P02088	Hbb-b1	Hemoglobin subunit beta-1	0.043	7.706
Q61646	Hp	Haptoglobin	0.012	2.706
Q91X72	Hpx	Hemopexin	0.002	7.352

Section S2.3. DNA damage, male UNT vs male WT

Protein	Code	Name	SWATH	
			P_value	FC
P17751	Tpi1	Triosephosphate isomerase	0.0225	16.6208
P0CG50	Ubb	Polyubiquitin-B	0.0088	39.5216
P61089	Ube2n	Ubiquitin-conjugating enzyme E2 N	0.0386	3.9333

Section S3.1. Nucleocytoplasmic traffic of macromolecules and e histones, male UNT vs Mtol WT.

Protein	Code	Name	SWATH	
			P_value	FC
P10922	H1-0	Histone H1.0	2.09E-06	15.7769
P43275	H1-1	Histone H1.1	0.0124	2.6155
P15864	H1-2	Histone H1.2	0.0158	14.7239
P43274	H1-4	Histone H1.4	0.0263	32.3093
Q3THW5	H2az2	Histone H2A.V	0.0332	10.3191
P62806	H4c1	Histone H4	0.0276	22.0122

Section S3.2. Ribosomes, male UNT vs male WT.

Protein	Code	Name	SWATH	
			P_value	FC
P97461	Rps5	40S ribosomal protein S5	0.0178	9.3185
P62270	Rps18	40S ribosomal protein S18	0.0358	3.2491
Q9CZX8	Rps19	40S ribosomal protein S19	0.0317	1.9008
P60867	Rps20	40S ribosomal protein S20	0.0305	2.0351
P62852	Rps25	40S ribosomal protein S25	0.0425	5.4571
P35979	Rpl12	60S ribosomal protein L12	0.0281	6.4042

Section S3.3. Proteosome, male UNT vs male WT

Protein	Code	Name	SWATH	
			P_value	FC
P97372	Psme2	Proteasome activator complex subunit 2	0.0009	2.0607

Section S3.4. Vesicular transport, male UNT vs male WT

Protein	Code	Name	SWATH	
			P_value	FC
P35278	Rab5c	Ras-related protein Rab-5C	0.0026	4.2406
P46638	Rab11b	Ras-related protein Rab-11B	0.0040	2.4525
Q9CYZ2	Tpd52l2	Tumor protein D54	0.0383	7.4121

Section S3.5. Lysosome, male UNT vs male WT

Protein	Code	Name	SWATH	
			P_value	FC
Lysosome membrane				

P24668	M6pr	Cation-dependent mannose-6-phosphate receptor	0.0047	3.2882
P11438	Lamp1	Lysosome-associated membrane glycoprotein 1	0.0098	2.7102
P16045	Lgals1	Galectin-1	0.0179	9.2213
P16110	Lgals3	Galectin-3	0.0260	3.2915
Lysosomal enzymes				
Q9WV54	Asah1	Acid ceramidase	0.0527	0.1694

Section S3.6. Cytoskeleton, male UNT vs male WT

Protein	Code	Name	SWATH	
			P_value	FC
P0DP28	Calm3	Calmodulin-3	0.0244	0.1649
P68134	Acta1	Actin, alpha skeletal muscle	0.0527	0.1694
P10833	Rras	Ras-related protein R-Ras	0.0029	2.6307
Q62234	Myom1	Myomesin-1	0.0376	0.4040
P97457	Myfpl	Myosin regulatory light chain 2, skeletal muscle isoform	0.0188	9.3929
P51667	Myl2	Myosin regulatory light chain 2, ventricular/cardiac muscle isoform	0.0371	5.4004
Q61792	Lasp1	LIM and SH3 domain protein 1	0.0050	3.2746
P20801	Tnnc2	Troponin C, skeletal muscle	0.0301	0.2303
Q80X90	Flnb	Filamin-B	0.0466	0.4545
P47754	Capza2	F-actin-capping protein subunit alpha-2	0.0335	2.2634
P26039	Tln1	Talin-1	0.0309	1.7377
Q9QUI0	Rhoa	Transforming protein RhoA	0.0080	4.4309
Q9ESM6	Gdpd2	Glycerophosphoinositol inositolphosphodiesterase GDPD2	0.0305	0.0173
Q9JM76	Arpc3	Actin-related protein 2/3 complex subunit 3	0.0012	3.9724
Q9CPW4	Arpc5	Actin-related protein 2/3 complex subunit 5	0.0179	9.5173
Q9D898	Arpc5l	Actin-related protein 2/3 complex subunit 5-like protein	0.0088	3.0402

Section S4.1. Extracellular matrix, hyaluronan and proteoglycans, male UNT vs male WT

Protein	Code	Name	SWATH	
			P_value	FC
Proteoglycans				
Hyalectans				
Q9QUP5	Hapln1	Hyaluronan and proteoglycan link protein 1	0.0140	1.7277
Small Leucine-rich proteoglycans				
Class I				
P28654	Dcn	Decorin	0.0005	3.0871
Class II				

P50608	Fmod	Fibromodulin	0.0450	6.9376
P51885	Lum	Lumican	0.0175	20.7626
Q9JK53	Prelp	Prolargin	0.0174	15.2827
O35367	Kera	Keratocan	0.0383	12.1981
Class IV				
O55226	Chad	Chondroadherin	0.0446	2.0533

Section S4.2. Extracellular matrix, Collagens, male UNT vs male WT

Protein	Code	Name	SWATH	
			P_value	FC
Fibrillar collagen				
P11087	Col1a1	Collagen alpha-1(I) chain	0.0137	24.3614
Q01149	Col1a2	Collagen alpha-2(I) chain	0.0360	70.2564
P28481	Col2a1	Collagen alpha-1(II) chain	0.0120	2.1669
Q61245	Col11a1	Collagen alpha-1(XI) chain	0.0187	12.4530
Q64739	Col11a2	Collagen alpha-2(XI) chain	0.0161	10.7360

Section S4.3. Extracellular matrix: matricellular proteins, male UNT vs male WT

Protein	Code	Name	SWATH	
			P_value	FC
Extracellular matrix targeting				
P35441	Thbs1	Thrombospondin-1	0.0309	10.4717
Q05793	Hspg2	Basement membrane-specific heparan sulfate proteoglycan core protein	0.0346	2.8603

Section S4.4. Extracellular matrix: extracellular matrix proteases, male UNT vs male WT

Protein	Code	Name	SWATH	
			P_value	FC
Extracellular matrix proteases				
Metzincin				
P41245	Mmp9	Matrix metalloproteinase-9	0.0125	5.5616
Plasminogen/plasmin system				

P07759	Serpina3k	Serine protease inhibitor A3K	0.0413	1.0905
Q9D154	Serpinb1a	Leukocyte elastase inhibitor A	0.0264	2.9116
P97298	Serpinf1	Pigment epithelium-derived factor	0.0469	9.2363
Cathepsins proteases				
Aspartic proteases				
P18242	Ctsd	Cathepsin D	0.0489	5.5031
Cysteine proteases				
P49935	Ctsh	Pro-cathepsin H	0.0226	3.8861

Section S4.5. Extracellular matrix: extracellular matrix receptor, male UNT vs male WT

Protein	Code	Name	SWATH	
			P_value	FC
Integrins				
Q61738	Itga7	Integrin alpha-7	0.0140	1.9660
P05555	Itgam	Integrin alpha-M	0.0038	3.7745
P43406	Itgav	Integrin alpha-V	0.0018	3.5819
P11835	Itgb2	Integrin beta-2	0.0266	2.6178
P29533	Vcam1	Vascular cell adhesion protein 1	0.0375	0.3312

Section S4.6. Extracellular matrix: discoidin domain receptors, male UNT vs male WT

Protein	Code	Name	SWATH	
			P_value	FC
P60766	Cdc42	Cell division control protein 42	0.0287	2.5824

Section S4.7. Extracellular matrix: CD receptors, male UNT vs male WT

Protein	Code	Name	SWATH	
			P_value	FC
P31428	Dpep1	Dipeptidase 1	0.0258	79.0435

Section S5.1. Other proteins of interest, male UNT vs male WT

Protein	Code	Name	SWATH	
			P_value	FC
O70622	Rtn2	Reticulon-2	0.0388	4.6641
Q9QXC1	Fetub	Fetuin-B	0.0420	11.0009
P07724	Alb	Albumin	0.0332	27.5579

Q6P8J7	Ckmt2	Creatine kinase S-type, mitochondrial	0.0354	8.4023
