# Supplementary material for: Morquio A Syndrome: Identification of Differential Patterns of Molecular Pathway Interactions in Bone Lesions
Source: Int J Mol Sci. 2024 Mar 12;25(6):3232. doi: 10.3390/ijms25063232 (PMC10970612; doi:10.3390/ijms25063232)
Supplement: Supplementary file 1 [file ijms-25-03232-s001.zip › Supplementary table S8.pdf]

Protein	Code	Name	Interac Ldha	Interac Ldhb	UNT vs WT FC
P12787	COX5A	Cytochrome c oxidase subunit 5A, mitochondrial	Yes	No	5.6382
P97457	MLRS	Myosin regulatory light chain 2, skeletal muscle isoform	Yes	No	9.3929
P62897	CYC	Cytochrome c, somatic	Yes	No	4.9513
P0DP28	CALM3	Calmodulin-3	Yes	No	0.1649
Q9CQQ7	AT5F1	ATP synthase F(0) complex subunit B1, mitochondrial	Yes	No	2.1821
P17742	PPIA	Peptidyl-prolyl cis-trans isomerase A	Yes	No	0.4396
P34884	MIF	Macrophage migration inhibitory factor	Yes	No	7.8439
P10126	EF1A1	Elongation factor 1-alpha 1	Yes	No	3.1414
P07724	ALBU	Albumin	Yes	No	27.5579
Q91VD9	NDUS1	NADH-ubiquinone oxidoreductase 75 kDa subunit, mitochondrial	No	Yes	2.0976
Q8BH95	ECHM	Enoyl-CoA hydratase, mitochondrial	No	Yes	0.4641
P11404	FABPH	Fatty acid-binding protein, heart	No	Yes	33.7581
P05202	AATM	Aspartate aminotransferase, mitochondrial	Yes	Yes	7.8887
P06745	G6PI	Glucose-6-phosphate isomerase	Yes	Yes	3.2287
P05064	ALDOA	Fructose-bisphosphate aldolase A	Yes	Yes	18.7808
Q9D0F9	PGM1	Phosphoglucomutase-1	Yes	Yes	1.9249
P17182	ENOA	Alpha-enolase	Yes	Yes	0.2793
P35700	PRDX1	Peroxiredoxin-1	Yes	Yes	3.4018
Q61171	PRDX2	Peroxiredoxin-2	Yes	Yes	8.0147
Q9D051	ODPB	Pyruvate dehydrogenase E1 component subunit beta, mitochondrial	Yes	Yes	0.3913
Q8K2B3	SDHA	Succinate dehydrogenase [ubiquinone] flavoprotein subunit, mitochondrial	Yes	Yes	2.6715
Q9CQA3	SDHB	Succinate dehydrogenase [ubiquinone] iron-sulfur subunit, mitochondrial	Yes	Yes	14.5768
Q9D6R2	IDH3A	Isocitrate dehydrogenase [NAD] subunit alpha, mitochondrial	Yes	Yes	3.4270
P08249	MDH2	Malate dehydrogenase, mitochondrial	Yes	Yes	19.0472
P17751	TPI1	Triosephosphate isomerase	Yes	Yes	16.6208
Q91YT0	NDUV1	NADH dehydrogenase [ubiquinone] flavoprotein 1, mitochondrial	Yes	Yes	3.9303
Q9DB20	ATPO	ATP synthase subunit O, mitochondrial	Yes	Yes	6.7524
O08749	DLDH	Dihydrolipoyl dehydrogenase, mitochondrial	Yes	Yes	1.9254
Q9WUB3	PYGM	Glycogen phosphorylase, muscle form	Yes	Yes	0.3059
