# Supplementary figures and images for: Morquio A Syndrome: Identification of Differential Patterns of Molecular Pathway Interactions in Bone Lesions
Source: Int J Mol Sci. 2024 Mar 12;25(6):3232. doi: 10.3390/ijms25063232 (PMC10970612; doi:10.3390/ijms25063232)

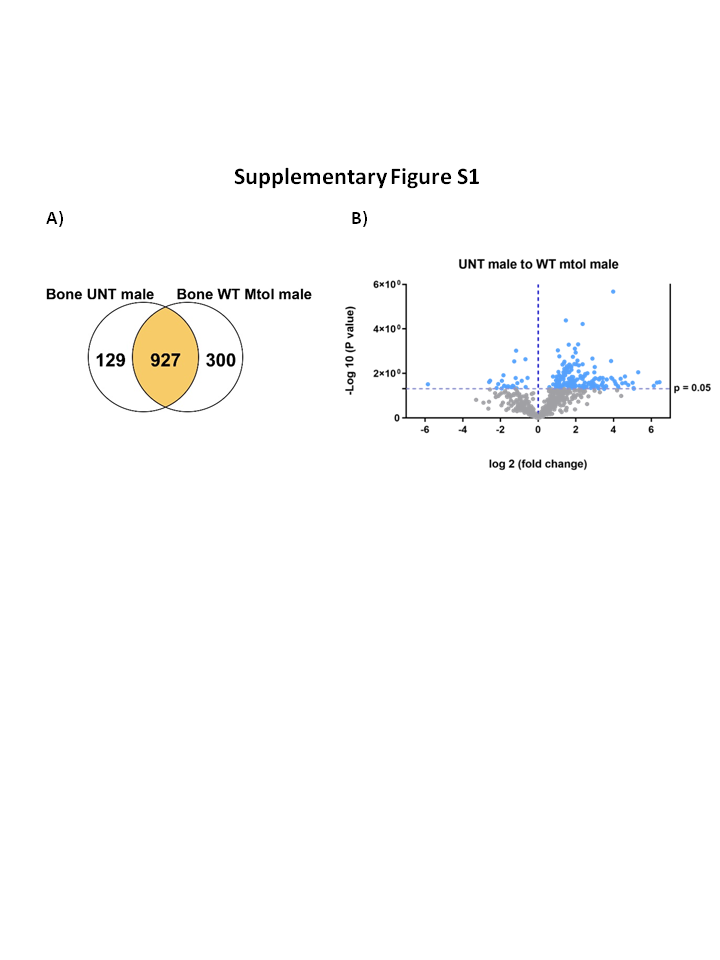

Supplement: Supplementary file 1 [file ijms-25-03232-s001.zip › Supplementary Figure S1.TIF]
